# Red blood cell transfusion Results

**DOI:** 10.1186/cc12304

**Published:** 2013-03-19

**Authors:** R Miadda, C Colognesi, L Hajjar, M Sundin, L Camara, S Zeferino, F Bergamin, A Leme, V Guimaraes, F Galas

**Affiliations:** 1 Heart Institute, São Paulo, Brazil

## Introduction

Cardiac surgeries are associated with high rates of bleeding and consequently with high rates of allogeneic transfusion. Patients with left ventricular dysfunction usually present a higher incidence of complications after cardiac surgery, including low output syndrome and organ dysfunction. It is a matter of controversy whether this subgroup of patients need higher hemoglobin levels to optimize oxygen delivery. We aimed to evaluate the association of transfusion with worse outcomes in patients with left ventricular dysfunction.

## Methods

A prospective cohort study was conducted in 215 patients undergoing cardiac surgery, presenting ejection fraction <35%. We recorded baseline characteristics, intraoperative data, transfusion requirement and severe postoperative complications as need for reoperation, acute kidney injury, arrhythmia, severe sepsis, septic shock, bleeding, stroke, and death during 30 days. We performed univariate analysis using baseline, intraoperative and postoperative variables associated with severe postoperative complications. Selected variables (*P <*0.10) were included in a forward stepwise multiple logistic regression model to identify predictive factors of a combined endpoint including 30-day mortality and severe complications.

## Results

Two hundred and fifteen patients were included in the study. The mean ejection fraction was 31%. From all patients, 129 (54.4%) presented the combined endpoint. The incidence of RBC transfusion was higher in the group with complications compared with the group without complications both intraoperative and postoperative, *P <*0.001. Patients with complications were older, had lower initial hemoglobin levels and had higher EuroSCORE. At multivariate analysis, age (OR = 1.07, 95% CI = 1.013 to 1.137, *P *= 0.016) and perioperative RBC transfusion (OR = 7.78, 95% CI = 2.13 to 28.41, *P *= 0.002) were independent predictors of severe postoperative complications after cardiac surgery.

## Conclusion

In patients with left ventricular dysfunction, red blood transfusion after cardiac surgery is a risk factor for worse outcome including 30-day mortality.

